# Patterns of Dietary Supplement Use for Weight Loss Among U.S. Adults With Obesity: NHANES 2007–2020

**DOI:** 10.1155/jobe/6761414

**Published:** 2026-04-08

**Authors:** Ligang Liu, Heqing Tao, Hao Zhao, Jie Wang, Kunkun Wang, Guang Xiong, Milap C. Nahata

**Affiliations:** ^1^ Institute of Therapeutic Innovations and Outcomes, College of Pharmacy, The Ohio State University, Columbus, Ohio, USA, osu.edu; ^2^ Department of Pharmacy Practice and Administration, College of Pharmacy, Western University of Health Sciences, Pomona, California, USA, westernu.edu; ^3^ Department of Gastroenterology, The First Affiliated Hospital of Guangzhou Medical University, Guangzhou Medical University, Guangzhou, China, gzhmc.edu.cn; ^4^ Department of General Surgery, Peking Union Medical College Hospital, Chinese Academy of Medical Sciences & Peking Union Medical College, Beijing, China, cams.ac.cn; ^5^ China Academy of Chinese Medical Sciences, Xiyuan Hospital, Beijing, China, xyhospital.com; ^6^ School of Medicine, University of Washington, Seattle, Washington, USA, washington.edu; ^7^ Department of Geriatric Endocrinology and Metabolism, The First Affiliated Hospital of Guangxi Medical University, Nanning, Guangxi, China, gxmu.edu.cn; ^8^ College of Medicine, The Ohio State University, Columbus, Ohio, USA, osu.edu

**Keywords:** dietary supplements, obesity, patterns, prevalence, weight loss

## Abstract

**Background:**

Dietary supplements are commonly marketed for weight loss, yet contemporary national data remain limited on the prevalence of use, types of products used, and whether use is provider‐recommended among U.S. adults with obesity.

**Methods:**

We conducted a cross‐sectional analysis using data from the National Health and Nutrition Examination Survey (NHANES) from 2007 to 2020. Participants included adults with obesity. They were asked about behaviors on dietary supplements for weight loss. We estimated survey‐weighted prevalence overall and by survey cycle, assessed linear trends over time, and used survey‐weighted multivariable logistic regression to examine associations with age, sex, race/ethnicity, education, and poverty–income ratio. Among users, we described whether supplement use was recommended by healthcare providers and summarized supplement categories.

**Results:**

Among 27,116 adults with obesity, 1706 reported using dietary supplements for weight loss. The survey‐weighted prevalence of weight‐loss supplement use was 5.9% and varied across survey cycles (*p* for trend = 0.003). Compared with adults aged 20–39 years, odds of use were lower among those aged 40–59 years (adjusted odds ratio [aOR] 0.60, 95% CI 0.45–0.80) and ≥ 60 years (aOR 0.15, 95% CI 0.11–0.22). Females had higher odds of use than males (aOR 1.46, 95% CI 1.13–1.87). Education was positively associated with use, whereas poverty–income ratio was not significantly associated. The use differed by race/ethnicity, with non‐Hispanic White adults having lower odds of use compared with Hispanic adults. Among users, 81% reported use without medical advice, and it was more common among males and younger adults. The most frequently used supplements included multivitamin–minerals (28.8%), botanical products (20.0%), and single vitamins (15.2%).

**Conclusions:**

Approximately 6% of U.S. adults with obesity reported using dietary supplements for weight loss, most without clinician recommendation and with significant demographic differences. Routine clinical assessment of supplement use and counseling regarding limited efficacy and potential safety concerns are warranted.

## 1. Introduction

Obesity is an escalating global health crisis that substantially increases the risk of multiple chronic diseases, including type 2 diabetes, cardiovascular diseases, and cancers [1]. Over 42% of adults in the United States (US) are classified as having obesity, with projections indicating that nearly 50% of adults will have obesity by 2030 [2, 3]. This increased prevalence imposes significant health concerns and associated economic burdens, amounting to billions of dollars annually [4, 5].

Lifestyle interventions remain the cornerstone of obesity treatment. However, the physiological adaptations of weight loss can promote weight gain, leading to only modest weight loss [6]. Pharmacotherapy has been used for individuals unable to achieve significant weight loss through lifestyle modifications alone [7]. Antiobesity medications are designed to reduce appetite, enhance satiety, or decrease nutrient absorption [8]. Despite their effectiveness, their use may be limited by adverse events, such as gastrointestinal disturbances as well as high cost and access barriers for some agents, such as glucagon‐like peptide‐1 receptor agonists (GLP‐1RAs) [9]. Furthermore, the difficulty in sustaining weight loss after treatment discontinuation poses significant challenges to the long‐term success of these interventions [10]. Given the concerns of potential adverse effects as well as limited insurance coverage, many individuals seek alternative options with increased interest in dietary supplements for weight loss [9, 11].

Dietary supplements are widely marketed as “safe” aids for weight loss, often claiming benefits, such as appetite suppression, metabolic enhancement, or increased fat oxidation [12]. However, weight‐loss dietary supplements raise important safety concerns, particularly when used without clinician supervision. Surveillance reports and reviews have documented adverse events associated with weight‐loss and sports supplements, including hepatotoxicity, cardiovascular complications, and products adulterated with pharmacologically active ingredients [13]. These risks may be underestimated by consumers, in part because many assume supplements undergo premarket safety and efficacy review by the regulatory agency (US Food and Drug Administration) similar to prescription medications [14].

Despite widespread use, contemporary nationally representative data describing weight‐loss dietary supplement use specifically among adults with obesity remain limited. In 1998, 11.3% of adults with obesity reported using nonprescription weight‐loss products [15], but these findings predate major changes in the supplement marketplace, marketing channels, and obesity epidemiology. In addition, there is limited evidence describing whether use is primarily self‐initiated versus clinician‐recommended, and which supplement types are most frequently used among adults with obesity. Updated national estimates can inform provider knowledge, clinical counseling, and public health strategies to support safe, evidence‐based weight management.

Therefore, this study aimed to (1) estimate the contemporary prevalence of dietary supplement use for weight loss among U.S. adults with obesity; (2) examine demographic and socioeconomic factors associated with use; (3) characterize whether supplement use was self‐initiated or recommended by healthcare providers; and (4) describe the types of supplements used using National Health and Nutrition Examination Survey (NHANES) 2007–2020 data. We hypothesized that weight‐loss dietary supplement use among U.S. adults with obesity would vary by sociodemographic characteristics, and most use was self‐initiated rather than provider‐recommended.

## 2. Methods

### 2.1. Study Design and Population

This cross‐sectional study used data from NHANES collected between 2007 and 2020. NHANES is a nationally representative, multistage, probability‐based survey of the U.S. civilian, noninstitutionalized population [16]. Detailed descriptions of the NHANES sampling design and methodology are provided by the National Center for Health Statistics and were followed in this analysis. Because the 2019–2020 cycle was truncated due to the COVID‐19 pandemic, the Centers for Disease Control and Prevention combined 2017–2018 and 2019–March 2020 into a single 2017–2020 prepandemic dataset. This study used publicly available, de‐identified data from NHANES. NHANES protocols were approved by the National Center for Health Statistics Research Ethics Review Board, and written informed consent was obtained from all participants. Because this analysis involved secondary analysis of de‐identified public‐use data, it was exempt from additional institutional review board review.

We included adults aged ≥ 20 years who were not pregnant and had anthropometric measurements available from the NHANES Examination component (body measures). Obesity was defined using examination‐based measures as body mass index (BMI) ≥ 30 kg/m^2^ and/or abdominal obesity (waist circumference > 102 cm in men or > 88 cm in women). Height, weight, and waist circumference were measured by trained technicians using standardized protocols.

To distinguish population‐level prevalence estimation from characterization of supplement users, we used a two‐step analytic approach. In Step 1, we identified the analytic denominator as all eligible adults with obesity. In Step 2, within the obesity denominator, we identified participants who reported dietary supplement use for weight loss using either of two NHANES sources: (1) the Weight History Questionnaire (WHQ), in which participants reported taking any nonprescription supplements to lose weight in the past 12 months (WHD080J = 32), and/or (2) the Dietary Supplement Use data (DSQIDS), in which at least one supplement taken in the prior 30 days was reported as used specifically for weight loss (DSQ128N = 23).

**FIGURE 1:**
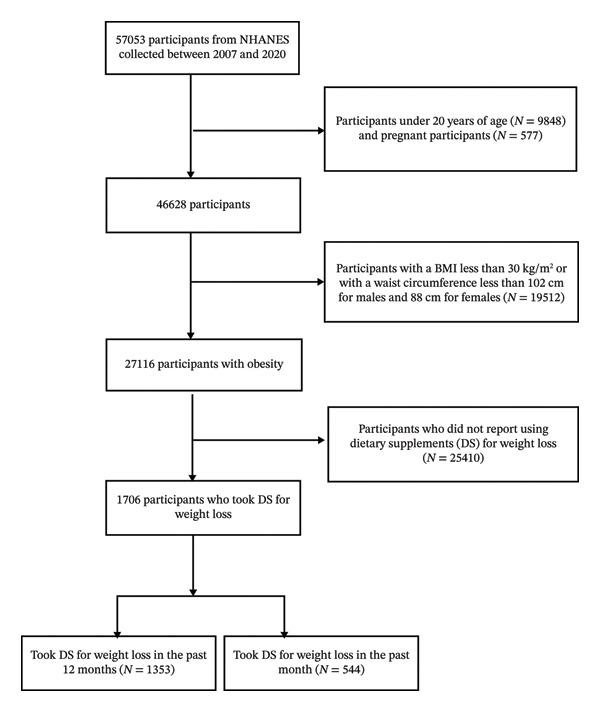
Flowchart of inclusion and exclusion of the study population. Note. Participants meeting either criterion were classified as users (*n* = 1706). Among users, 1353 reported the use of nonprescription supplements to lose weight in the past 12 months, 544 reported using dietary supplements in the past 30 days for weight loss, and 191 met both criteria.

As shown in Figure 1, among 57,053 NHANES participants, we excluded those aged < 20 years (*N* = 9848) and pregnant participants (*N* = 577), leaving 46,628 eligible adults. We then excluded adults without obesity (*N* = 19,512), resulting in 27,116 adults with obesity (Step 1). Within this obesity denominator, 1706 participants were classified as weight‐loss dietary supplement users (Step 2); 1353 met the 12‐month criterion, 544 met the 30‐day criterion, and 191 met both.

### 2.2. Measures and Covariates

We collected data from multiple modules of the NHANES database, including Demographics, Examination, Questionnaire, and Dietary Supplement modules. The Demographics module provided data on participant characteristics, including age, gender, race, family income‐to‐poverty ratio (PIR), and education level. Participants were categorized into three age groups: young adults (20–39 years), middle‐aged adults (40–59 years), and older adults (60 years and older). Race and ethnicity were classified into four categories: Hispanic, non‐Hispanic White, non‐Hispanic Black, and other races. Education level was classified into three categories: less than high school, high school graduate or equivalent, and some college or higher. Income levels were grouped into three categories: low‐income (PIR ≤ 1), middle‐income (1 < PIR < 4), and high‐income (PIR ≥ 4) [17].

Weight‐loss behaviors were assessed using WHQ items, including whether participants reported trying to lose weight during the past 12 months. Dietary supplement use was assessed via DSQIDS, which captures supplements taken in the past 30 days and the reported purpose for each supplement. Participants were also asked whether their use of supplements was self‐initiated or recommended by a healthcare provider (DSQ124).

### 2.3. Supplement Type Classification

Dietary supplements recorded in the dietary module were classified into predefined categories, including multivitamin–mineral products (containing three or more vitamins and one or more minerals), multimineral products (containing two or more minerals without vitamins), multivitamin products (containing two or more vitamins without minerals), botanical products (containing one or more botanical ingredients without vitamins or minerals), and specific nutrient products focused primarily on individual components, such as calcium, omega‐3 fatty acids, fiber, probiotics, prebiotics, amino acids, chondroitin, glucosamine, melatonin, and specific B‐complex and single vitamins.

Botanical supplements were further categorized by primary ingredients, such as apple cider vinegar, coconut oil, caffeine, ginger, green tea, green coffee, garcinia cambogia, and raspberry ketone. Supplements that did not fit any prespecified botanical ingredient subtype or represented heterogeneous plant‐derived/specialty bioactives were grouped as “other.” This category included products, such as konjac/glucomannan (e.g., Lipozene), seaweed/algae‐derived products (e.g., kelp and “green oxygen” formulations), citrus/tea concentrates, and other botanicals or bioactives (e.g., turmeric/curcumin, garlic extract, cinnamon, resveratrol, and fucoxanthin), as well as specialty compounds used in weight‐management products (e.g., carnitine, phytosterols, palmitoleic acid, and 5‐HTP) and mixed‐ingredient formulations. Products with insufficient detail to assign a specific subtype were retained in the “other” category.

### 2.4. Statistical Analysis

Six continuous cycles (2007–2008, 2009–2010, 2011–2012, 2013–2014, 2025–2016, and 2017–March 2020 prepandemic) were combined for analysis. Variance estimates, expressed as percentages with standard errors (SEs), were generated using the Taylor series linearization in accordance with NHANES guidelines [18]. Survey weights, strata, and primary sampling units were incorporated to account for the complex sampling design and to produce nationally representative estimates.

Survey‐weighted prevalence estimates of dietary supplement use for weight loss were calculated using the obesity denominator (Step 1). Descriptive characteristics of users, supplement types, and provider recommendation analyses were conducted among weight‐loss supplement users (Step 2). Group differences in survey‐weighted proportions were assessed using design‐based tests appropriate for complex survey data. Time trends were evaluated using survey‐weighted regression models with the NHANES cycle. Survey‐weighted multivariable logistic regression models were used to identify independent predictors of weight‐loss dietary supplement use among adults with obesity, adjusting for demographic covariates; results are reported as adjusted odds ratios (aORs) with 95% confidence intervals. All analyses were conducted using R (Version 4.3.1) with the “survey” and “srvyr” packages. A two‐sided *p* value < 0.05 was considered statistically significant.

## 3. Results

### 3.1. Prevalence and Trends Over Time

**FIGURE 2:**
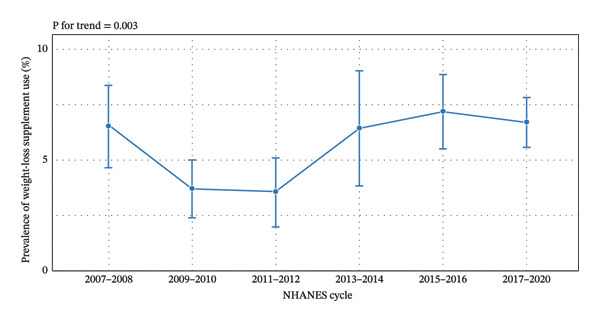
Trends in the prevalence of dietary supplement use for weight loss among U.S. adults with obesity, 2007–2020. Note. Points represent survey‐weighted prevalence estimates for each NHANES cycle; error bars indicate standard errors. The 2017–2020 cycle represents the combined 2017–2018 and 2019–March 2020 prepandemic dataset.

Among 27,116 adults with obesity, 1706 participants were classified as weight‐loss dietary supplement users. Using adults with obesity as the denominator, the survey‐weighted prevalence of weight‐loss supplement use was 5.9%. Patient characteristics are summarized in Table 1. In survey‐weighted trend analysis, weight‐loss supplement use changed significantly over time (*p* for trend = 0.003). Prevalence fluctuated across survey cycles, starting at 6.5% in 2007–2008, declining to 3.5% in 2009–2010 and 3.6% in 2011–2012, then increased to 6.4% in 2013–2014, peaked at 7.1% in 2015–2016, and stabilized at 6.6% during 2017–2020 (Figure 2). Cycle‐specific estimates are presented in Table 2.

**TABLE 1:** Baseline characteristics of adults with obesity who reported dietary supplement use for weight loss, NHANES 2007–2020 (*N* = 1706).

Characteristic	Overall (*n* = 1706)	2007–2008 (*n* = 132)	2009–2010 (*n* = 144)	2011–2012 (*n* = 145)	2013–2014 (*n* = 300)	2015–2016 (*n* = 340)	2017–2020 (*n* = 645)
Age group, y							
20–39	570; 38.5 (3.0)	54; 43.1 (6.7)	66; 48.7 (7.8)	72; 51.3 (11.5)	104; 41.3 (7.0)	108; 31.8 (5.1)	166; 35.6 (5.6)
40–59	768; 45.9 (3.0)	52; 43.4 (7.5)	59; 43.6 (9.0)	45; 35.5 (9.0)	141; 45.7 (7.7)	181; 59.3 (5.6)	290; 40.9 (5.5)
≥ 60	368; 15.6 (2.2)	26; 13.4 (4.4)	19; 7.7 (2.7)	28; 13.2 (4.7)	55; 13.0 (4.4)	51; 8.9 (2.1)	189; 23.4 (5.1)
Sex							
Men	510; 26.3 (2.6)	44; 27.0 (5.7)	58; 39.6 (7.6)	34; 17.8 (3.6)	102; 32.5 (4.0)	74; 25.4 (6.4)	198; 22.9 (4.7)
Women	1196; 73.8 (2.6)	88; 73.0 (5.7)	86; 60.4 (7.6)	111; 82.3 (3.6)	198; 67.6 (4.0)	266; 74.6 (6.4)	447; 77.1 (4.7)
Race/ethnicity							
Hispanic	410; 16.8 (2.1)	41; 14.8 (4.1)	47; 20.1 (8.5)	27; 15.7 (6.5)	54; 12.5 (3.8)	99; 14.6 (3.3)	142; 20.0 (4.4)
Non‐Hispanic White	603; 58.9 (3.2)	56; 67.2 (5.4)	60; 62.8 (8.7)	53; 62.7 (8.8)	127; 63.6 (5.6)	112; 59.5 (7.2)	195; 53.0 (6.6)
Non‐Hispanic Black	502; 15.2 (1.9)	29; 10.8 (2.7)	32; 13.5 (4.6)	54; 16.9 (3.7)	77; 14.8 (4.4)	93; 13.9 (3.7)	217; 17.1 (3.7)
Other	191; 9.2 (2.1)	6; 7.2 (4.0)	5; 3.6 (1.4)	11; 4.8 (1.9)	42; 9.1 (3.4)	36; 12.0 (6.9)	91; 9.9 (3.2)
Education level							
Less than high school	143; 5.0 (0.7)	13; 6.8 (1.9)	18; 6.3 (2.2)	10; 4.6 (2.5)	23; 6.2 (2.3)	38; 4.7 (0.9)	41; 4.1 (1.1)
High school/equivalent	336; 20.3 (2.6)	29; 27.0 (8.0)	30; 22.5 (6.8)	31; 20.2 (5.1)	52; 15.6 (4.4)	61; 14.7 (4.5)	133; 24.0 (5.4)
Some college or more	1227; 74.8 (2.6)	90; 66.2 (6.7)	96; 71.2 (7.2)	104; 75.2 (5.6)	225; 78.2 (4.5)	241; 80.6 (4.1)	471; 71.9 (5.7)
Family income‐to‐poverty ratio (PIR)							
≤ 1.0	238; 10.1 (1.1)	12; 7.5 (2.8)	25; 10.8 (3.1)	29; 17.6 (5.2)	50; 10.0 (2.4)	32; 5.1 (1.2)	90; 12.0 (1.9)
1.01–3.99	922; 51.6 (3.6)	68; 52.4 (9.0)	73; 41.1 (10.7)	71; 56.0 (6.7)	178; 61.5 (5.8)	195; 47.9 (7.8)	337; 50.1 (7.2)
≥ 4.0	444; 38.4 (3.7)	47; 40.1 (9.4)	41; 48.2 (10.7)	38; 26.4 (8.7)	70; 28.5 (6.4)	94; 47.0 (8.3)	154; 38.0 (7.3)

*Note:* Values are shown as unweighted *n*, weighted % (SE).

Abbreviation: SE = standard error.

**TABLE 2:** Prevalence of weight‐loss dietary supplement use among U.S. adults with obesity across NHANES cycles, by demographic and socioeconomic characteristics (*N* = 1706).

Characteristic	Overall (*n* = 1706)	2007–2008 (*n* = 132)	2009–2010 (*n* = 144)	2011–2012 (*n* = 145)	2013–2014 (*n* = 300)	2015–2016 (*n* = 340)	2017–2020 (*n* = 645)
Age group, y							
20–39	570; 13.1^∗^ (1.1)	54; 14.7 (2.8)	66; 9.8 (2.6)	72; 10.2 (2.4)	104; 15.9 (2.9)	108; 14.0 (3.1)	166; 13.4 (1.9)
40–59	768; 7.7 (0.8)	52; 6.6 (1.6)	59; 4.2 (1.0)	45; 3.2 (1.2)	141; 8.3 (3.0)	181; 13.3 (1.7)	290; 8.2 (1.5)
≥ 60	368; 1.9 (0.3)	26; 2.3 (0.7)	19; 0.6 (0.2)	28; 1.0 (0.5)	55; 1.7 (0.6)	51; 1.2 (0.4)	189; 3.2 (0.6)
Sex							
Men	510; 4.6 (0.5)	44; 5.0 (1.1)	58; 4.1 (1.2)	34; 1.8 (0.6)	102; 6.5 (1.8)	74; 5.3 (1.4)	198; 4.5 (0.9)
Women	1196; 6.6^∗^ (0.4)	88; 7.3 (1.2)	86; 3.4 (0.7)	111; 4.4 (1.0)	198; 6.4 (1.3)	266; 8.1 (1.3)	447; 7.8 (0.7)
Race/ethnicity							
Hispanic	410; 10.5^∗^ (0.9)	41; 11.5 (2.6)	47; 8.1 (1.9)	27; 6.6 (1.1)	54; 9.5 (1.9)	99; 11.2 (2.7)	142; 12.2 (1.7)
Non‐Hispanic White	603; 4.5 (0.4)	56; 5.5 (0.9)	60; 2.9 (0.7)	53; 2.8 (0.9)	127; 5.1 (1.4)	112; 5.4 (1.1)	195; 4.7 (0.8)
Non‐Hispanic Black	502; 11.5^∗^ (1.0)	29; 8.3 (1.4)	32; 6.2 (2.1)	54; 7.1 (1.3)	77; 11.8 (2.8)	93; 14.1 (2.2)	217; 14.4 (2.2)
Other	191; 10.2^∗^ (2.2)	6; 12.5 (7.4)	5; 3.1 (1.6)	11; 3.4 (1.8)	42; 13.3 (4.9)	36; 15.2 (8.1)	91; 10.2 (2.7)
Education level							
Less than high school	143; 3.1 (0.4)	13; 3.0 (1.0)	18; 1.7 (0.6)	10; 1.4 (0.5)	23; 3.9 (1.2)	38; 4.4 (1.2)	41; 4.1 (0.9)
High school/equivalent	336; 5.4 (0.7)	29; 6.8 (2.3)	30; 3.6 (1.1)	31; 3.6 (1.1)	52; 4.5 (1.5)	61; 5.8 (1.9)	133; 6.4 (1.4)
Some college or more	1227; 6.5 (0.5)	90; 7.2 (1.0)	96; 4.1 (1.0)	104; 3.8 (1.0)	225; 7.4 (1.7)	241; 7.8 (0.9)	471; 7.0 (1.0)
Income‐to‐poverty ratio (PIR)							
≤ 1.0	238; 7.2 (0.8)	12; 6.6 (2.2)	25; 5.1 (1.9)	29; 6.3 (2.0)	50; 6.6 (1.3)	32; 4.9 (0.8)	90; 10.2 (2.3)
1.01–3.99	922; 6.6 (0.5)	68; 7.2 (1.5)	73; 3.4 (1.2)	71; 3.9 (0.7)	178; 8.0 (2.0)	195; 8.2 (1.4)	337; 7.3 (0.8)
≥ 4.0	444; 5.4 (0.6)	47; 6.1 (1.5)	41; 4.2 (1.0)	38; 2.3 (0.9)	70; 4.9 (1.3)	94; 7.2 (1.5)	154; 5.9 (1.3)

*Note:* Values are presented as unweighted *n*; weighted % (SE). The unweighted *n* shown in each cell reflects the number of participants with obesity who reported weight‐loss supplement use in that subgroup/cycle.

Abbreviation: SE = standard error.

^∗^indicates *p* < 0.05 for overall differences across categories within the characteristic; statistical tests were not performed separately within each NHANES cycle.

### 3.2. Demographic Differences in Use

Females were significantly more likely to take supplements for weight loss compared to males (6.6% vs 4.6%, *p* < 0.001). Prevalence differed by age group (*p* < 0.001), with the highest use among adults aged 20–39 years (13.1%), followed by 40–59 years (7.7%) and ≥ 60 years (1.9%). Use also differed by race/ethnicity (*p* < 0.001), with higher prevalence among non‐White participants compared with non‐Hispanic White participants. No significant differences were observed across education or PIR categories in unadjusted analyses (Table 2).

### 3.3. Multivariable Survey‐Weighted Logistic Regression

After adjustment for age group, sex, race/ethnicity, education, and PIR, weight‐loss supplement use remained independently associated with several sociodemographic factors. Compared with adults aged 20–39 years, odds of use were lower for 40–59 years (aOR 0.60, 95% CI 0.45–0.80) and substantially lower for ≥ 60 years (aOR 0.15, 95% CI 0.11–0.22). Females had higher odds of use than males (aOR 1.46, 95% CI 1.13–1.87). Education was positively associated with use: Compared with less than high school, high school/equivalent was associated with higher odds (aOR 1.97, 95% CI 1.27–3.04) and some college or higher with still higher odds (aOR 2.26, 95% CI 1.65–3.10). PIR was not significantly associated with use (PIR 1.01–3.99: aOR 1.21, 95% CI 0.94–1.56; PIR ≥ 4.0: aOR 0.98, 95% CI 0.73–1.32). Associations differed across race/ethnicity categories: Compared with Hispanic participants, non‐Hispanic White participants had lower odds of use (aOR 0.50, 95% CI 0.37–0.68), whereas other comparisons were not statistically significant (Table 3).

**TABLE 3:** Survey‐weighted multivariable logistic regression of dietary supplement use for weight loss among U.S. adults with obesity, NHANES 2007–2020 (*N* = 1706).

Characteristic	Adjusted OR	95% CI
Age group (ref: 20–39 years)		
40–59 years	0.6	0.45–0.80
≥ 60 years	0.15	0.11–0.22
Sex (ref: men)		
Women	1.46	1.13–1.87
Race/ethnicity (ref: Hispanic)		
Non‐Hispanic White	0.5	0.37–0.68
Non‐Hispanic Black	1.1	0.82–1.47
Other	1.01	0.57–1.79
Education level (ref: less than high school)		
High school/equivalent	1.97	1.27–3.04
Some college or more	2.26	1.65–3.10
Income‐to‐poverty ratio (PIR) (ref: ≤ 1.0)		
1.01–3.99	1.21	0.94–1.56
≥ 4.0	0.98	0.73–1.32

*Note:* Results are from a survey‐weighted multivariable logistic regression model including age group, sex, race/ethnicity, education level, and PIR. Reference categories are shown in parentheses.

Abbreviations: CI = confidence interval, OR = odds ratio.

### 3.4. Provider Recommendation Versus Self‐Initiated Use

Among weight‐loss supplement users, 19.3% reported that their supplement use for weight loss was recommended by a healthcare provider, whereas 80.7% reported self‐initiated use. Provider‐recommended use was more common among adults aged ≥ 60 years (31.0%) than among younger adults (13.7% for 20–39 years and 19.9% for 40–59 years; *p* = 0.016) and was more common among females than males (21.7% vs 12.5%; *p* = 0.012). Provider recommendation did not differ significantly by race/ethnicity, education, or PIR (Table 4).

**TABLE 4:** Provider‐recommended dietary supplement use for weight loss among adults with obesity who reported weight‐loss supplement use, NHANES 2007–2020 (*N* = 304).

Characteristic	Unweighted *N*	Weighted % (SE)
Overall	304	19.3 (2.0)
Age (years)		
20–39	64	13.7 (2.3)
40–59	141	19.9 (3.9)
≥ 60	99	31.0 (5.0)^∗^
Sex		
Men	73	12.5 (2.7)
Women	231	21.7 (2.5)^∗^
Race/ethnicity		
Hispanic	63	13.5 (2.9)
Non‐Hispanic White	107	20.7 (3.1)
Non‐Hispanic Black	97	17.9 (2.4)
Other	37	24.2 (7.8)
Education level		
Less than high school	22	16.0 (3.8)
High school/equivalent	63	21.4 (3.8)
Some college or more	219	19.0 (2.7)
Income‐to‐poverty ratio (PIR)		
≤ 1.0	47	17.8 (3.4)
1.01–3.99	153	17.7 (2.2)
≥ 4.0	80	21.3 (5.2)

Abbreviation: SE = standard error.

^∗^indicates *p* < 0.05 for overall differences across categories within the characteristic.

### 3.5. Types of Supplements Used

Among weight‐loss supplement users, multivitamin–mineral products were the most commonly reported supplement type (28.8%), followed by botanicals (20.0%), single vitamins (15.2%), calcium (9.8%), omega‐3 fatty acids (6.5%), and single minerals (5.1%) (Figure 3(a)). Among single vitamins, vitamin D was most frequently reported (5.3%), followed by vitamin B12 (3.4%) and vitamin C (3.2%). Among botanical products, multiherbal formulations were the most common (3.6%), followed by Garcinia cambogia (2.1%), ginger (1.2%), green coffee bean (1.2%), raspberry ketone (0.9%), and green tea (0.8%). Less frequently reported botanical ingredients included coconut oil (0.6%), apple cider vinegar (0.3%), and acai berry extract (0.3%). In addition, “other” category (8.0%) included heterogeneous products not captured by prespecified ingredient subtypes, including mixed botanical/specialty formulations and products with insufficient detail for more specific classification (Figure 3(b)).

FIGURE 3Types of dietary supplements used for weight loss among U.S. adults with obesity (NHANES 2007–2020) (*N* = 544). (a) Broad dietary supplement categories. (b) Detailed dietary supplement classification. Note: Estimates are based on the NHANES Dietary Supplement Use 30‐day file and reflect supplements reported as taken in the past 30 days among adults with obesity. Percentages are survey‐weighted and account for the NHANES complex sampling design. Error bars indicate standard errors (SE). “Others” includes less frequently reported botanical/specialty products, mixed‐ingredient formulations not captured by predefined categories, and products with insufficient detail for more specific classification.

**Figure:**
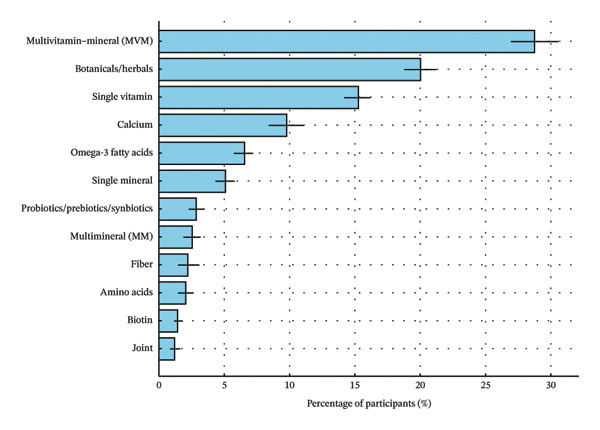
(a)

**Figure:**
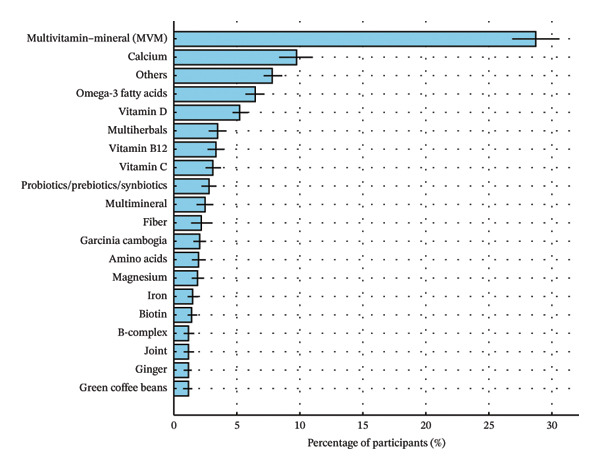
(b)

## 4. Discussion

In this nationally representative analysis of NHANES 2007–2020, 5.9% of U.S. adults with obesity reported using dietary supplements for weight loss. The use differed significantly by age, sex, and race/ethnicity, and most use was self‐initiated rather than recommended by a healthcare provider. These findings provide updated national estimates of weight‐loss supplement use among adults with obesity, describe the types of products used, and quantify the extent to which use occurred without clinical guidance.

The prevalence observed in our study was lower than prior population‐based estimates. Earlier surveys reported weight‐loss supplement use of 11.3% in 1998 and 8.7% in 2002, with 16.1% among individuals trying to lose weight [15, 19]. Another study conducted in 2005–2006 indicated that one‐third of participants who had made a serious attempt to lose weight reported ever using a dietary supplement for weight loss at some point [20]. Differences across studies likely reflect variation in definitions and recall windows. Earlier studies assessed supplement use for weight loss over 2 years, allowing participants to report any nonprescription product use at any time during that timeframe. In contrast, our definition combined NHANES supplement‐level reporting of weight‐loss intent for products used in the prior 30 days with an additional participant‐level item capturing any past 12‐month nonprescription supplement use for weight loss.

We observed significant variation over time, with lower prevalence during 2009–2012 and higher prevalence in subsequent cycles. These observed variations may reflect broader contextual factors during that period, such as recession‐related financial constraints [21]. Prevalence increased after 2011, and the post‐2011 increase is directionally consistent with industry reports indicating continued growth in U.S. dietary supplement sales and with national health expenditure analyses showing faster growth in U.S. health spending [22, 23].

Several demographic patterns were consistent with prior literature. Weight‐loss supplement use was more common among younger adults and women, and differences were observed across racial/ethnic groups. Another national survey also found that dietary supplement use was more prevalent among women compared to men, those aged 25–34 years, and African Americans and Hispanics [20]. Similar trends were observed in a study conducted in Poland, where a higher proportion of women and younger individuals reported using dietary supplements for weight loss [24]. Potential explanations include differences in weight‐related norms, body image pressures, perceived benefits, and marketing exposure, as well as cultural influences and differences in access to health information and healthcare resources [25, 26].

A key finding was that most weight‐loss supplement use occurred without provider recommendation. About 80.7% of users reported self‐initiated use, with men and younger adults being more prone to this practice. Prior work suggests that many users do not disclose supplement use to healthcare professionals [19]. Most people mistakenly believed that dietary supplements were subject to premarket safety and efficacy approvals by the regulatory agencies [20]. In addition, online sources may contribute to misinformation about efficacy and safety [27]. Males were more likely to disregard doctors’ recommendations than females [28]. Older people were more likely to engage in patient‐centered interactions with their physicians than younger adults because of their trust in doctors, shared decision‐making process, and their relationships with providers [29, 30]. These patterns underscore the need for clinicians to routinely ask about supplement use during obesity management and to counsel patients on limited efficacy evidence, possible drug–supplement interactions, and potential harms. Importantly, because serious adverse events related to weight‐loss supplements are frequently linked to adulteration or contamination with pharmacologically active compounds, improving regulatory oversight and product surveillance is essential rather than placing the burden on patients to verify product safety.

Americans spend over $2 billion annually on weight‐loss dietary supplements [31]. Costs and insurance coverage vary by product type. Common supplements, such as multivitamin–mineral products, are often relatively inexpensive, whereas many products marketed specifically for weight loss, particularly multi‐ingredient botanical formulations, can be more costly and are typically purchased out‐of‐pocket. The complexity of these multi‐ingredient products may also increase variability in formulation and quality and may heighten the potential for drug–supplement interactions.

The supplements reported by users in our study included both broad “general health” products (multivitamin–mineral products and single vitamins) and botanicals marketed for weight management. Regular consumption of vitamins may be associated with lower body weight and fat mass while increasing relative resting metabolic rate by decreasing appetite [32]. A previous study found that people believed that multivitamin use was important for their health among individuals who tried to lose weight [33]. Vitamin D might regulate adipocyte lipid metabolism and triglyceride storage, improving metabolic health and reducing body weight [34]. The claims for proposed mechanisms of action of herbal products can vary for each supplement, including stimulating energy expenditure, modulating carbohydrate metabolism, increasing satiety, increasing fat oxidation or decreasing fat synthesis, blocking absorption of fat, eliminating excessive body water, and enhancing mood [35]. Garcinia cambogia could affect appetite [36]. Any caffeine‐containing dietary supplement including green coffee beans might contribute to appetite suppression and stimulate thermogenesis [37]. Although some supplements, particularly multivitamin/multimineral products, may help individuals meet micronutrient needs or reduce nutrient gaps during calorie‐restricted dieting, they should not be interpreted as effective weight‐loss therapies. Overall, there is no conclusive evidence that any specific dietary supplement produces clinically meaningful reductions in body weight [38]. Current guidelines do not endorse dietary supplement use in managing obesity, and they emphasize comprehensive lifestyle interventions as the primary strategies for managing obesity [39, 40].

Given the high prevalence of self‐initiated weight‐loss supplement use among adults with obesity, clinicians should routinely ask about dietary supplement use during obesity care and counsel patients on the limited evidence for efficacy and the potential for harm [41]. This is particularly important because patients may not volunteer supplement use and may assume these products undergo premarket safety and efficacy review comparable to prescription medications. Accordingly, patient counseling should be paired with stronger regulatory oversight, improved postmarketing surveillance, and consumer education. Given the continued use of these unregulated products and the documented risk of adverse events, particularly from adulterated botanical supplements, clinicians, policymakers, and public health organizations should strengthen regulatory oversight, improve patient education, and promote evidence‐based strategies for obesity management. In clinical practice, routine screening for supplement use may help identify potential safety concerns and provide opportunities to counsel patients on proven strategies for weight management.

### 4.1. Strengths and Limitations

The study had several strengths, including the use of nationally representative data from the NHANES, which offers a comprehensive view of dietary supplement use among U.S. adults with obesity. The study also examined usage patterns across various demographic factors, such as age, sex, and race, providing deeper insights into differences in supplement use. Additionally, the analysis of longitudinal data allowed for the pattern of usage over time. However, the study also had limitations. The assessment of dietary supplement use was restricted to the past 30 days, which could underestimate the prevalence of use over the past year. The effectiveness of dietary supplements for weight management could not be determined due to the lack of data. Third, supplement use is self‐reported and may be subject to recall bias. Self‐report may also introduce misclassification of product identity and purpose and social desirability bias. These biases could affect prevalence estimates and subgroup comparisons in either direction. Furthermore, the same dietary supplements could be used for multiple reasons, not solely for weight loss, which complicates the interpretation of their intended use.

## 5. Conclusions

In this nationally representative analysis of NHANES 2007–2020, 5.9% of U.S. adults with obesity reported using dietary supplements for weight loss, with significant variation by age, sex, and race/ethnicity. Use was predominantly self‐initiated, and fewer than one in five users reported taking supplements for weight loss based on a healthcare provider recommendation. The most commonly used products included multivitamin–mineral supplements and botanicals, including multi‐ingredient formulations that may pose safety concerns. These findings highlight the importance of routine clinical screening for supplement use during obesity care, patient counseling on limited efficacy and potential harms, and strengthened regulatory oversight and surveillance to reduce risks associated with weight‐loss dietary supplements.

## Author Contributions

Ligang Liu contributed to the study conceptualization, investigation, and methodology and drafted the manuscript; he also participated in manuscript review and editing. Heqing Tao curated the data, conducted the formal analyses, validated the findings, and assisted with manuscript review. Hao Zhao contributed to the investigation and provided project resources. Jie Wang contributed to the investigation and methodology and developed the study visualizations. Kunkun Wang provided resources and contributed to manuscript review and editing. Guang Xiong contributed to conceptualization and formal analysis and assisted with manuscript writing and revision. Milap C. Nahata acquired funding, supervised the study, and contributed to manuscript review and editing.

## Funding

No funding was received for this manuscript.

## Conflicts of Interest

The authors declare no conflicts of interest.

## Data Availability

The data that support the findings of this study are available from the corresponding author upon reasonable request.
